# Author Correction: Surrogate “Level-Based” Lagrangian Relaxation for mixed-integer linear programming

**DOI:** 10.1038/s41598-023-30358-9

**Published:** 2023-03-01

**Authors:** Mikhail A. Bragin, Emily L. Tucker

**Affiliations:** 1 Department of Electrical and Computer Engineering, University of Connecticut, 371 Fairfield Way, U-4157, Storrs, 06269 CT USA; 2 Department of Industrial Engineering, Clemson University, 271 Freeman Hall, Clemson, SC 29634 USA

Correction to: *Scientific Reports* 10.1038/s41598-022-26264-1, published online 27 December 2022

This Article contained errors in the algorithm in the Results section, under the subheading ‘Algorithm: Pseudocode’.

In “Input”, that the initial level value needs to be an overestimate of the optimal dual values was omitted. In addition, $q^{max}$ has been initialized as a small value.

In line 1, the $y^{feas}$ was omitted.

In line 5, the equation number was incorrectly given as ‘(20)’.

In line 7, the subscript "0" for $q^{max}$ has been removed.

In line 13, the subscript "j" for $q^{max}$ has been removed, and $q^{max}$ has been set as a small value.

In line 15, the $y^{feas}$ was omitted, and what *f* is has been clarified.

The original algorithm appears below.
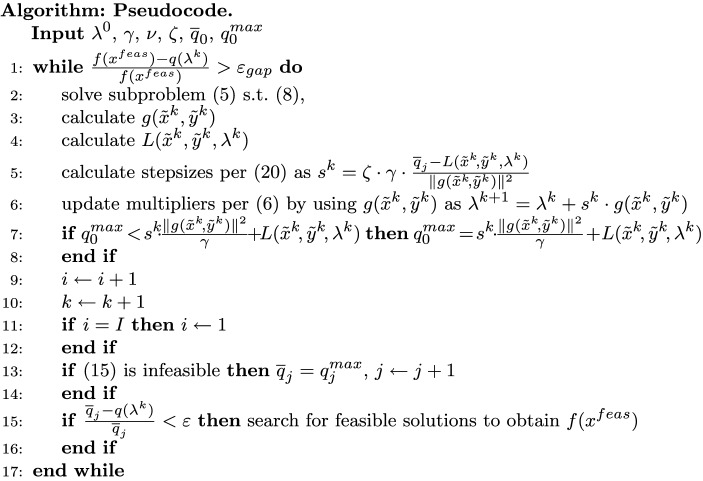


The original Article has been corrected.

