# Supplementary material for: Surrogate “Level-Based” Lagrangian Relaxation for mixed-integer linear programming
Source: Sci Rep. 2022 Dec 27;12:22417. doi: 10.1038/s41598-022-26264-1 (PMC9794831; doi:10.1038/s41598-022-26264-1)
Supplement: Supplementary file 1 — Supplementary Information. [file 41598_2022_26264_MOESM1_ESM.pdf]

4 SUPPLEMENTARY INFORMATION.

4.1 Methods: Previous Methods for Non-smooth Optimization and for MILP

The 1990s: The Subgradient-Level Method. The Subgradient-Level method, developed by Goffin and Kiwiel[24], overcomes the unavailability of the knowledge about the optimal value needed to compute Polyak's stepsize (7) by adaptively adjusting a "level" estimate based on the detection of "sufficient descent" of the function and "oscillation" of solutions.

In terms of the problem (3), the procedure of the method is explained as follows: the "level" estimate $q^k_{lev} = q^{k_j}_{rec} + \delta_j$ is used in place of the optimal dual value $q(\lambda^*)$, where q^k_{rec} is the best dual value ("record objective value") obtained up to an iteration k, and δ_j is an adjustable parameter with j denoting the jth update of q^k_{lev}. The main premise behind this is when δ_j is "too large," then multipliers will exhibit oscillations while traveling a significant (predefined) distance R without improving the "record" value. In this case, the parameter δ_j is updated as $\delta_{j+1} = \beta \cdot \delta_j$ with $\beta = \frac{1}{2}$. On the other hand, if δ_j is such that the dual value is sufficiently increased: $q(\lambda^k) \geq q^k_{lev} + \tau \cdot \delta_j$, with $\tau = \frac{1}{2}$, then the parameter δ_j is unchanged and the distance traveled by multipliers is reset to 0 to avoid premature reduction of δ_j by β in future iterations.

The Early 2000s: Incremental Subgradient Methods [25,27]. The main idea of the Incremental Subgradient method is to improve convergence by solving a subproblem i before updating multipliers. After one subgradient component is updated, rather than updating all the multipliers "at once," within the incremental subgradient methods, multipliers are updated "incrementally." After the ith subgradient component is calculated, the multipliers are incrementally updated as

$$\psi^k_i = \psi^k_{i-1} + s^k \cdot \left(A_i x^k_i - \beta_i\right). \tag{26}$$

Here β_i are the vectors such that $\sum_{i=1}^{I} \beta_i = b$, for example, $\beta_i = \frac{b}{I}$. Only after all i subproblems are solved, are the multipliers "fully" updated as

$$\lambda^{k+1} = \psi^k_I. \tag{27}$$

Convergence results of the Subgradient-Level method[24] have been extended for the Incremental Subgradient method and proved. Variations of the method were proposed with β and τ belonging to an interval $[0, 1]$ rather than being equal to $\frac{1}{2}$. Moreover, to improve convergence, rather than using constant R, a sequence of R_l was proposed such that $\sum_{l=1}^{\infty} R_l = \infty$. While the method reduces the effort by solving one subproblem as a time, in order to compute the "level" values, the "record" dual value is required; in order to obtain dual values, all subproblems need to be solved optimally without updating the multipliers.

The 2010s: The Surrogate Lagrangian Relaxation Method [26]. Convergence of the method is based on the "contraction mapping" concept. Namely, within the method, distances between multipliers at consecutive iterations are required to decrease, i.e.,

$$\|\lambda^{k+1} - \lambda^k\| = \alpha_k \cdot \|\lambda^k - \lambda^{k-1}\|, \quad 0 \leq \alpha_k < 1. \tag{28}$$

Based on (10), the stepsizing formula has been derived:

$$s^k = \alpha_k \cdot \frac{s^{k-1}\|g(\tilde{x}^{k-1})\|}{\|g(\tilde{x}^k)\|}. \tag{29}$$

Moreover, a specific formula to set α_k has been developed to guarantee convergence:

$$\alpha_k = 1 - \frac{1}{M \cdot k^{1-\frac{1}{k^r}}}, \ M \geq 1, \ 0 \leq r \leq 1. \tag{30}$$

Surrogate Absolute-Value Lagrangian Relaxation [28]. Aiming to simultaneously guarantee convergence while ensuring a fast reduction of constraint violations and preserving the linearity of the original MILP problem, the Surrogate Absolute-Value Lagrangian Relaxation (SAVLR) method was developed. Within the method, the following dual problem is considered:

$$\max_{\lambda} \{q_\rho(\lambda) : \lambda \in \Omega \subset \mathbb{R}^m\}, \tag{31}$$

where

$$q_\rho(\lambda) = \min_{(x,y)} \left\{ \sum_{i=1}^{I} \left((c_i^x)^T x_i + (c_i^y)^T y_i\right) + \lambda^T \cdot \left(\sum_{i=1}^{I} A_i^x x_i + \sum_{i=1}^{I} A_i^y y_i - b\right) + \right.$$
$$\left. \rho \cdot \| \sum_{i=1}^{I} A_i^x x_i + \sum_{i=1}^{I} A_i^y y_i - b\|_1, \{x_i, y_i\} \in \mathcal{F}_i, i = 1, \ldots, I \right\}. \tag{32}$$

The above minimization involves l_1-absolute-value piece-wise linear penalties, which efficiently penalize constraint violations and are exactly linearizable thereby enabling the use of MILP solvers. `

4.2 Example *Generalized Assignment Problems.*

The mathematical formulation of GAP is:

$$\min_{x_{i,j}} \sum_{i=1}^{I} \sum_{j=1}^{J} g_{i,j} x_{i,j}, \quad x_{i,j} \in \{0,1\}, \quad g_{i,j} \geq 0, \tag{33}$$

$$s.t. \quad \sum_{i=1}^{I} a_{i,j} x_{i,j} \leq b_j, \quad j = 1, \ldots, J, \quad a_{i,j} \geq 0, \ b_j \geq 0, \tag{34}$$

$$\sum_{j=1}^{J} x_{i,j} = 1, \quad j = 1, \ldots, J. \tag{35}$$

The objective (33) is to minimize the total assignment cost by deciding which job i is to be assigned to which machine j; if job i is assigned to machine j, then $x_{i,j} = 1$ and $x_{i,j} = 0$, otherwise. Constraints (34) ensure that the total amount of time required by all jobs to be processed on machine j should not exceed the total machine's available time b_j. The assignment constraints (35) ensure that each job is assigned to only one machine. Within SLBLR, constraints (35) are relaxed.

4.3 Example *Stochastic job-shop scheduling with the consideration of scrap and rework.*

For the job-shop scheduling example, to create a difficult testing case, data from Hoitomt et al.[24] are considered and several parameters are modified. Namely, 16 jobs: Jobs 1 through 6 and 118 through 127 originally with one operation, are modified by adding 5 more operations, with processing times generated randomly from a range [1, 5] based on a discrete uniform distribution. Moreover, to create a case mimicking "labor shortage", the machine capacity of certain machine types is reduced by 2, and the resulting machine capacities are $M = \{1, 1, \mathbf{2}, 2, 1, 1, \mathbf{2}, 1, 1, \mathbf{4}, 1, 1, 1, 1, 2, 1, 1\}$, with

modified capacities shown in bold. The due dates and the number of operations per job are shown in Table S1. Several due dates, originally negative, are modified. Other parameters such as processing times are shown in Table S2.

Table S1. Data for Example *Stochastic job-shop scheduling with the consideration of scrap and rework.*

i	d_i	J_i	i	d_i	J_i	i	d_i	J_i	i	d_i	J_i
1	10	6	33	37	2	65	-81	1	97	14	1
2	10	6	34	37	2	66	37	1	98	14	1
3	9	6	35	159	2	67	37	1	99	37	1
4	61	6	36	8	1	68	71	2	100	-1	1
5	61	6	37	12	1	69	88	2	101	2	1
6	-3	6	38	20	1	70	18	1	102	-4	1
7	-85	3	39	40	1	71	8	2	103	4	1
8	10	1	40	62	1	72	31	2	104	7	1
9	18	1	41	84	1	73	35	2	105	53	2
10	145	3	42	35	1	74	36	1	106	95	2
11	145	3	43	35	1	75	39	2	107	132	2
12	-169	2	44	157	1	76	40	2	108	174	2
13	-114	1	45	157	1	77	42	2	109	218	2
14	-123	1	46	157	1	78	44	2	110	261	2
15	91	3	47	157	1	79	31	2	111	304	2
16	91	3	48	35	1	80	35	2	112	28	2
17	91	3	49	35	1	81	36	2	113	2	1
18	91	3	50	7	1	82	39	2	114	2	1
19	91	3	51	-53	1	83	40	2	115	111	1
20	91	3	52	70	1	84	42	2	116	111	1
21	-65	3	53	3	1	85	44	2	117	-6	2
22	-51	3	54	-4	1	86	71	1	118	17	6
23	-47	1	55	12	1	87	71	1	119	-1	6
24	-35	1	56	29	2	88	88	1	120	-27	6
25	2	1	57	132	1	89	88	1	121	-6	6
26	6	1	58	-22	1	90	71	2	122	-6	6
27	-47	2	59	13	1	91	81	2	123	1	6
28	38	1	60	-10	1	92	11	1	124	56	6
29	38	1	61	12	1	93	8	1	125	78	6
30	161	1	62	12	1	94	8	1	126	98	6
31	-45	1	63	6	6	95	11	1	127	19	6
32	-53	1	64	35	1	96	8	1			

4.4 Example *Multi-stage pharmaceutical scheduling.*

The data and formulation for the case example of multi-stage pharmaceutical scheduling come from Kopanos et al.[13]. In the following, we use terminology and notation consistent with its original presentation. A set of product orders I are manufactured across a set of S stages of production. At each stage, product $i \in I$ is processed on exactly one unit $j \in J$, representing a machine. Each order $i \in I$ has a due date, δ_i. Deviations from this due date are penalized with a unit weight of w^+ (tardiness) and w^- (earliness). Setup times for each order have sequence-independent and sequence-dependent components. Sequence-independent setup times, π_i, are only based on the order $i \in i$. The sequence-dependent setup times, $\gamma_{s,i,i'}$, for order $i' \in I$ in stage $s \in S$ vary based on the order $i \in I$ it follows. The processing time of order $i \in I$ on unit $j \in J$ is given by $\tau_{i,j}$. A Big-M parameter M is defined as a sufficiently large number.

Binary assignment variables, $y_{j,i,s}$, indicate if unit $j \in J$ processes order $i \in I$ in stage $s \in S$. Binary precedence variables, $x_{j,i,i'}$, designate if order i is processed before $i' \in I$ when both i, i' are assigned to unit $j \in J$. The time that order $i \in I$ completes stage $s \in S$ is given by continuous variables, $c_{i,s}$. The tardiness and earliness of each order $i \in I$ are given by continuous variables, t_i and e_i, respectively.

$$\min_{t_i, e_i, y_{j,i,s}, x_{j,i,i'}} \sum_{i \in I} \{w^+ t_i + w^- e_i\}, \tag{36}$$

$$s.t. \quad e_i \geq \delta_i - c_{i,S_i^{last}}, \quad \forall i \in I, \tag{37}$$

Table S2. Data for Example *Stochastic job-shop scheduling with the consideration of scrap and rework.*

i	j	$p_{i,j}$	m	i	j	$p_{i,j}$	m	i	j	$p_{i,j}$	m	i	j	$p_{i,j}$	m
1	1	1	1	20	2	1	2	69	2	1	10	112	1	2	5
1	2	3	1	20	3	1	3	70	1	1	8	112	2	1	10
1	3	3	1	21	1	1	2	71	1	3	15	113	1	5	10
1	4	3	1	21	2	1	3	71	2	1	1	114	1	3	10
1	5	4	1	21	3	2	2	72	1	1	13	115	1	4	2
1	6	1	1	22	1	1	3	72	2	1	13	116	1	3	3
2	1	6	1	22	2	1	2	73	1	1	13	117	1	1	9
2	2	5	1	22	3	2	3	73	2	1	13	117	2	1	9
2	3	1	1	23	1	1	2	74	1	1	13	118	1	1	10
2	4	2	1	24	1	1	11	75	1	1	13	118	2	6	10
2	5	2	1	25	1	2	12	75	2	1	13	118	3	5	10
2	6	6	1	26	1	2	2	76	1	1	13	118	4	4	10
3	1	2	1	27	1	28	8	76	2	1	13	118	5	1	10
3	2	1	1	27	2	3	9	77	1	1	13	118	6	2	10
3	3	1	1	28	1	1	1	77	2	1	13	119	1	2	10
3	4	3	1	29	1	1	1	78	1	1	13	119	2	4	10
3	5	5	1	30	1	1	1	78	2	1	13	119	3	2	10
3	6	5	1	31	1	1	8	79	1	1	13	119	4	5	10
4	1	1	2	32	1	10	5	79	2	1	13	119	5	3	10
4	2	5	2	33	1	2	10	80	1	1	13	119	6	2	10
4	3	3	2	33	2	2	14	80	2	1	13	120	1	2	2
4	4	2	2	34	1	2	10	81	1	1	13	120	2	3	2
4	5	1	2	34	2	2	14	81	2	1	13	120	3	5	2
4	6	4	2	35	1	2	10	82	1	1	13	120	4	1	2
5	1	6	3	35	2	2	14	82	2	1	13	120	5	2	2
5	2	6	3	36	1	14	10	83	1	1	13	120	6	3	2
5	3	3	3	37	1	7	10	83	2	1	13	121	1	3	3
5	4	5	3	38	1	7	10	84	1	1	13	121	2	3	3
5	5	2	3	39	1	7	10	84	2	1	13	121	3	1	3
5	6	3	3	40	1	7	10	85	1	1	13	121	4	6	3
6	1	3	2	41	1	7	10	85	2	1	13	121	5	4	3
6	2	1	2	42	1	2	10	86	1	1	10	121	6	4	3
6	3	6	2	43	1	2	10	87	1	1	10	122	1	1	2
6	4	5	2	44	1	2	10	88	1	1	10	122	2	1	2
6	5	1	2	45	1	2	10	89	1	1	10	122	3	2	2
6	6	6	2	46	1	2	10	90	1	5	7	122	4	5	2
7	1	2	5	47	1	2	10	90	2	1	10	122	5	2	2
7	2	4	6	48	1	2	10	91	1	5	7	122	6	2	2
7	3	2	6	49	1	2	10	91	2	1	10	123	1	1	3
8	1	1	7	50	1	2	10	92	1	2	10	123	2	4	3
9	1	6	8	51	1	1	2	93	1	2	10	123	3	3	3
10	1	4	8	52	1	7	3	94	1	2	10	123	4	2	3
10	2	2	6	53	1	2	10	95	1	2	10	123	5	4	3
10	3	1	8	54	1	2	10	96	1	2	10	123	6	6	3
11	1	4	8	55	1	1	10	97	1	1	10	124	1	1	9
11	2	2	6	56	1	4	5	98	1	1	10	124	2	4	9
11	3	1	8	56	2	3	10	99	1	4	2	124	3	3	9
12	1	28	8	57	1	13	16	100	1	2	3	124	4	2	9
12	2	3	9	58	1	10	5	101	1	2	2	124	5	4	9
13	1	1	10	59	1	13	7	102	1	1	3	124	6	6	9
14	1	1	10	60	1	3	4	103	1	2	2	125	1	1	9
15	1	1	3	61	1	13	4	104	1	2	3	125	2	4	9
15	3	1	2	62	1	13	4	105	1	1	10	125	3	3	9
16	1	1	3	63	1	1	1	105	2	4	5	125	4	2	9
16	2	1	2	63	2	1	1	106	1	1	10	125	5	4	9
16	3	1	3	63	3	1	1	106	2	4	5	125	6	6	9
17	1	1	2	63	4	1	1	107	1	1	10	126	1	1	9
17	2	1	3	63	5	1	1	107	2	4	5	126	2	4	9
17	3	1	2	63	6	1	1	108	1	1	10	126	3	3	9
18	1	1	3	64	1	2	9	108	2	4	5	126	4	2	9
18	2	1	2	65	1	1	10	109	1	1	10	126	5	4	9
18	3	1	3	66	1	1	10	109	2	4	5	126	6	6	9
19	1	1	2	67	1	1	10	110	1	1	10	127	1	1	17
19	2	1	3	68	1	5	7	110	2	4	5	127	2	4	17
19	3	1	2	68	2	1	10	111	1	1	10	127	3	3	17
20	1	1	3	69	1	5	7	111	2	4	5	127	4	2	17

$$t_i \geq c_{i,S_i^{last}} - \delta_i, \quad \forall i \in I, \tag{38}$$

$$\sum_{j \in J} y_{j,i,s} = 1, \quad \forall i \in I, s \in S, \tag{39}$$

$$c_{i,s} \geq \sum_{j \in J} (\tau_{i,j} + \pi_i) y_{j,i,s}, \quad \forall i \in i, s = 1, \tag{40}$$

$$c_{i,s} - \sum_{j \in J} (\tau_{i,j} + \pi_i) y_{j,i,s} = c_{i,s-1}, \quad \forall i \in i, s \in S \setminus \{1\}, \tag{41}$$

$$c_{i,s} + \gamma_{s,i,i'} \leq c_{i',s} - \pi_{i'} - \tau_{i',j} + M(1 - x_{j,i,i'}) + M(2 - y_{j,i,s} - y_{j,i',s}), \quad \forall s \in S, j \in J, i, i' \in I : i' \geq i+1, \tag{42}$$

$$c_{i',s} + \gamma_{s,i',i} \leq c_{i,s} - \pi_i - \tau_{i,j} + M(1 - x_{j,i,i'}) + M(2 - y_{j,i,s} - y_{j,i',s}), \quad \forall s \in S, j \in J, i, i' \in I : i' \geq i+1, \tag{43}$$

$$y_{j,i,s}, x_{j,i,i'} \in \{0, 1\}, \quad \forall j \in J, i, i' \in I, s \in S, \tag{44}$$

$$t_i, e_i, c_{i,s} \geq 0, \quad \forall i \in I, s \in S. \tag{45}$$

The objective function (36) minimizes the total weighted deviations of orders from their due dates (referred to as in the original paper as "weighted lateness"). Constraints (37) and (38) record the earliness and tardiness, respectively, of each order $i \in I$. Constraints (39) require each order $i \in I$ to be assigned exactly one unit $j \in J$ in each stage $s \in S$. Constraints (40) ensure the first stage completion times of each order $i \in I$ are at least the sum of the independent setup and processing times on the assigned unit $j \in J$. Similar constraints (41) ensure subsequent stage completion times as a function of independent setup, processing, and completion times of the previous stage. Constraints (42)-(43) enforce the sequencing between orders $i, i' \in I$ for each unit $j \in J$ and each stage $s \in S$. Constraints (44)-(45) are standard domain constraints.

There are 17 units and 6 stages of production. The problem is run with both 30 and 60 product orders. Minor changes from the data provided by Kopanos et al.[13] are the incorporation of sequence-independent setup times and a non-optional stage three. We incorporated sequence-independent setup times by selecting the sequence-dependent setup times for one product in one stage, i.e., P01 in stage five. We generated sequence-dependent, stage three processing times for orders $i \in I$ that skipped stage three by randomly sampling from the two values given for the other products. To enforce the unallowable product-unit combinations, we set sufficiently large processing times where appropriate.

4.5 Proof of Corollary 1.

Proof. Define two predicates

$$A = \left\{ s^k < \gamma \cdot \frac{q(\lambda^*) - L(\tilde{x}^k, \tilde{y}^k, \lambda^k)}{\|g(\tilde{x}^k, \tilde{y}^k)\|^2} \right\}. \tag{46}$$

and

$$B = \left\{ \|\lambda^* - \lambda^{k+1}\| < \|\lambda^* - \lambda^k\| \right\}. \tag{47}$$

From Theorem 1 the following is true $A \Longrightarrow B$ and from Corollary 1 the following is true $\neg B \Longrightarrow \neg A$. It remains to prove that both assertions are equivalent. Taking negation of $A \Longrightarrow B$ leads to $\neg A \vee B$, and taking negation of $\neg B \Longrightarrow \neg A$ leads to $\neg(\neg B) \vee \neg A$, which simplifies to $B \vee \neg A$. □
